# Ethanol Inactivated Mouse Embryonic Fibroblasts Maintain the Self-Renew and Proliferation of Human Embryonic Stem Cells

**DOI:** 10.1371/journal.pone.0130332

**Published:** 2015-06-19

**Authors:** Boxian Huang, Song Ning, Lili Zhuang, Chunyan Jiang, Yugui Cui, Guoping Fan, Lianju Qin, Jiayin Liu

**Affiliations:** 1 School of Life Science and Technology, China Pharmaceutical University, Nanjing, 210038, China; 2 State Key Laboratory of Reproductive Medicine, Center of Clinical Reproductive Medicine, First Affiliated Hospital, Nanjing Medical University, Nanjing, 210029, China; 3 Department of Pediatrics, First Affiliated Hospital, Nanjing Medical University, Nanjing, Jiangsu Province, 210029, China; 4 Department of Human Genetics, David Geffen School of Medicine, UCLA, Los Angeles, California, 90095, United States of America; Baylor College of Medicine, UNITED STATES

## Abstract

Conventionally, mouse embryonic fibroblasts (MEFs) inactivated by mitomycin C or irradiation were applied to support the self-renew and proliferation of human embryonic stem cells (hESCs). To avoid the disadvangtages of mitomycin C and irradiation, here MEFs were treated by ethanol (ET). Our data showed that 10% ET-inactivated MEFs (eiMEFs) could well maintain the self-renew and proliferation of hESCs. hESCs grown on eiMEFs expressed stem cell markers of NANOG, octamer-binding protein 4 (OCT4), stage-specific embryonic antigen-4 (SSEA4) and tumour related antigen-1-81 (TRA-1-81), meanwhile maintained normal karyotype after long time culture. Also, hESCs cocultured with eiMEFs were able to form embryoid body (EB) *in vitro* and develop teratoma *in vivo*. Moreover, eiMEFs could keep their nutrient functions after long time cryopreservation. Our results indicate that the application of eiMEF in hESCs culture is safe, economical and convenient, thus is a better choice.

## Introduction

Human embryonic stem cells (hESCs) are pluripotent, can give rise to all tissue and cell types of the human body [1], thus have offered great promise for regenerative medicine, gene and cell therapies, and disease modeling[2]. It is great importance to keep hESC pluripotency in long term culture, thus the culture system is very crucial. To date, two types of hESC culture systems have been established: feeder-dependent[3] culture systems (FDCSs) and feeder-free culture systems (FFCSs)[4–6]. FFCSs are convenient in hESC culture, but expensive in that they require special cell-independent culture surfaces[7–17] and external growth factors[18–21]. Further more, most of FFCSs were tested only for 5–10 passages on supporting hESC proliferation[22], not guarantee for long term culture, thus the risk degree of spontaneous differentiation and karyotype aberrance for hESC might increase after several dozen passages culture[23]. Therefore, FDCS is still the first choice in basic research needing for long term culture of hESCs. In FDCSs, mouse embryonic fibroblasts (MEFs) are usually used as feeder layers to co-cultured with hESCs. MEFs have the function to supply the essential intrinsic regulators and environmental cues[24, 25], which are extremely important for regulating hESC growth, self-renewal and differentiation[26]. Generally, before co-culturing with hESCs, MEFs are inactivated by irradiation or mitomycin C (MC) to simplify the co-culture system and get maximum nutrition support. Irradiation can provide high-quality inactivated MEFs, but special machine and materials are required[27], which limit broad application of the method. There are concerns with MC-inactivated MEFs, too. First, it is time consuming. Second, MC is expensive. Third, residual MC may produce cytotoxicological effects on stem cell fate[28]. Thus, it is necessary to explore other safe, convenient, economical methods for preparing feeder cells to support hESCs growth. Recently, it is reported that formaldehyde (FA)- or glutaraldehyde (GA)-fixed MEFs can maintain the growth of mouse embryonic stem cells[29], monkey embryonic stem cells[29] and mouse induced pluripotent stem cells[30], suggesting that chemical-fixed approaches are choices for making feeder cells. However, culture systems based on chemical-fixed feeder layers is similar to FFCSs, thus can not guarantee for long term culture because they support hESC culture through the alike way. It is well verified that inactivated MEFs (after MC or irradiation treated) can provide full-scale nutritions for hESC self-renew and proliferation. Therefore, MEFs inactivated (not fixed) by healthy, cheap and convenient chemicals are advance choice.

Interestingly, it is reported that 70% ethanol could completely fix MEF and provide “live-cell” free substrate for stem cells[31]. There are also reports implicated that ethanol can inhibit cell proliferation through decreasing DNA synthesis[32, 33]. These studies suggest that ethanol might be used as MC to prepare inactivated MEFs. Ethanol shows several advantages. First, ethanol is compatible with human body at low concentration, thus it is safe to operators and hESCs in application. Second, ethanol is a stock chemical in worldwide biochemical and molecular laboratory, easy to get and convenient in application. Third, the price of ethanol is much lower than mitomycin C, thus money saving. Finally, there are reports showed that various types of human cells, such as human fetal liver stromal cells and human adult marrow cells can maintain the hESC proliferation[34, 35]. Thus ethanol-inactivated human-derived MEFs could be directly applied in clinical trials in the future.

In this study, different concentrations of ethanol were applied to treat MEFs. We found that 10% ethanol-inactivated MEFs (eiMEFs) were capable of supporting the long-term self-renew and proliferation of hESCs. Meanwhile, the pluripotency and normal karyotype of hESCs growing on eiMEFs were kept. Most importantly, similar to miMEFs and radiated-MEFs, eiMEFs can be cryopreserved in liquid N_2_ for a long time, then re-thawed and plated to support hESCs growth. Our results show that eiMEFs are applicable nutritive cells in hESCs mantenance. Our work will promote the hESCs-based research in future.

## Materials and Methods

### Isolation of MEFs

Pregnant ICR mice were purchased from Model Animal Research Center of Nanjing University, and embryos of 12.5-day old were isolated from the mice. Briefly, heads and all viscera of embryos were removed after embryos were separated from uterus. The remaining embryo were minced into pieces, digested with 0.25% trypsin/ ethylene diamine tetraacetic acid (EDTA: Invitrigen, USA), incubated in Dulbecco’s modified Eagle’s medium (DMEM: Invitrigen, USA) plus 10%FBS (Invitrigen, USA) at 37°C with 5% CO_2_. The Institutional Animal Care Committee of Nanjing Medical University approved the experimental protocol.

### Preparation of mitomycin C-inactivated MEFs

MEFs at passage 2–5 were inactivated with 10 μg/mL mitomycin C (Roche, USA) for 2.5–3 h. The cells were collected following digestion with 0.05% trypsin/EDTA, counted, and plated onto 0.1% gelatin-coated (Invitrigen, USA) dish or plate with the density of 2.5×10^4^/cm^2^.

### Preparation of ethanol inactevated MEFs

The culturing MEFs (at passage 2–5) were washed with phosphate buffer saline (PBS, Invitrogen, USA) and then were incubated in ethanol (ET) solutions (5%, 10%, 20% and 30%: Sigma, USA) at room temperature for 30 min. Subsequently, cells were washed three times with PBS, 10 mins per time. Finally, MEFs were enzymic digestion, plated onto 0.1% gelatin-coated dish or plate and used freshly, or freezing and stored in liquid nitrogen for future experiments.

### Cultivation of hESCs

Undifferentiated H9 hESCs from WiCell Research Institute (Madison, WI) were cultured as described previously[36] on MEF, respectively. The hESC culture medium is composed of DMEM/F12, 20% Knockout Serum, 1% Glutamin, 1% Nonessential Amino Acids, 0.1mM 2-mercaptoethanol, 1% penicillin streptomycin (all purchased from Invitrogen, USA), and 10 ng/mL basic fibroblast growth factor (bFGF: Peprotech,USA), at 37°C with 5% CO_2_, 20% O_2_. Medium was changed daily. After 5–7 days, hESC cells were digested for 10 min with 1mg/ml collagenase IV (Invitrogen, USA) and then plated onto new MEFs every 5–7 days.

### Alkaline phosphatase (AKP) activity assay

Alkaline Phosphatase Detection Kit (Vector Lab, USA) was selected to carry out alkaline phosphatase (AKP) staining of hESCs according to the manufacturer’s protocol.

### Counting living MEFs

Trypan blue was used to stain dead cells. Then living and dead cells were counted.

### Fluorescence activated cell sortor (FACS) analysis

To detect the expression of stem cell markers of NANOG (Abcam, USA), SSEA-4 (Abcam, USA), OCT4 (Abcam, USA), TRA-1-81 (Abcam, USA), hESCs were treated by trypsin-EDTA for 3 min and then blowed into single cell. Single hESCs were resuspended in 1% fetal bovine serum (FBS) diluted in PBS and then stained by the antibodies of SSEA-4, TRA-1-81 or its corresponding isotype control for 30 min at 4°C. Cytofix/Cytoperm Fixation/Permeabilization Solution Kit (BD, USA) was applied to stain OCT4 and NANOG following the manufacturer’s instruction. The stained cells were analyzed on fluorescence-activated cell sorter (BD, USA).

### Immunofluorescence staining

The primary antibodies of anti-OCT4, anti-SSEA4, anti-NANOG, and anti-TRA-1-81 (all from Chemicon, USA) were used to characterize hESCs. Briefly, cells cultured on coverslips were fixed with 4% PFA at room temperature for 10 min, permeated with 0.1% Triton X-100 (Sigma, USA)/Phosphate Buffer Solution (PBS) on ice for 10 min, and blocked with fresh 2% bovine serum albumin (BSA: Sigma, USA)/PBS at room temperature for 30 min. The treated hESCs were washed with PBS for 5 min and then incubated with primary antibodies over night at 4°C. After rinsed with PBS for 5 min, the hESCs were stained by Cy2-conjugated or FITC-conjugated secondary antibodies (Jackson Immunoresearch, West Grove) in dark for 30 min. The hESCs were mounted with 4', 6-diamidino-2-phenylindole (DAPI: Vector Lab, USA) after being washed with PBS for 5 min, and then photographed under fluorescence microscope (Nikon, Japan).

### Karyotype analysis

Standard G-band chromosome analysis was performed by the Medical Test Institute, Nanjing Medical University.

### RNA extraction and real-time polymerase chain reaction (PCR)

Total RNA was extracted using the QIAGEN RNeasy Mini Kit (QIAGEN). We performed first-strand cDNA synthesis using Superscript Reverse Transcriptase (Invitrogen). Total RNA was reverse-transcribed to cDNA using PrimeScript RT Reagent Kit (Takara, Japan) according to the manufacturer’s instructions. Quantitative real-time PCR was performed using Thermal Cycler Dice Real Time System (Takara, Japan) and SYBR Premix Ex Taq (Takara, Japan). Cycle time (Ct) values were obtained using the ABI PRISM 7900 Sequence Detection System and analysis software (Applied Biosystems). Each sample was quantified against its GAPDH transcript content. Experiments were repeated three times, and results are presented as fold change ± SD. The sequences of primers used in real-time PCR are listed in Table 1.

**Table 1:** Designations, sequences, and the sizes of real-time PCR amplicons.

Gene	Primer sequence from 5'-3'	Size (bp)
*NESTIN*	Forward *TTGCCTGCTACCCTTGAGAC*	145
	Reverse *GGGCTCTGATCTCTGCATCTAC*	
*SOX1*	Forward *CCTGTGTGTACCCTGGAGTTTCTGT*	180
	Reverse *TGCACGAAGCACCTGCAATAAGATG*	
*T*	Forward *CAGTGGCAGTCTCAGGTTAAGAAGGA*	120
	Reverse *CGCTACTGCAGGTGTGAGCAA*	
*CD31*	Forward *TCTATGACCTCGCCCTCCACAAA*	50
	Reverse *GAACGGTGTCTTCAGGTTGGTATTTCA*	
*AFP*	Forward *TTGACTGCAATTGAGAAACCCA*	71
	Reverse *AAGGCAGGTAGCTGGTTTTCTAAA*	
*SOX17*	Reverse *GTGGACCGCACGGAATTTG*	94
	Forward *GGAGATTCACACCGGAGTCA*	
*GAPDH*	Forward *GAAGGTCGGAGTCAACGGATTT*	223
	Reverse *CTGGAAGATGGTGATGGGATTTC*	

### Induce hESCs differentiation into dopaminergic neuron

H9 cells were induced differentiation into dopaminergic neurons according previous report[37]. Briefly, hESC colonies were detached with 1mg/ml collagenase IV (Invitrogen, USA) and 1X dispase (Invitrogen, USA) and cultured in ultralow-attachment dishes containing the hESC culture medium without bFGF and feeder for 7 days to form embryoid bodies (EBs). Then EBs were cultured in neural precursor cells (NPCs) induced medium (composed of DMEM-F12 and N2) for 4 days. The resulted NPCs were expanded for another 3 days in expansion medium (composed of DMEM-F12, N2 and bFGF-2). In order to form spherical neural masses (SNMs), neural rosettes and neural tube-like structures those presented in neural expansion culture were mechanically isolated and cultured in ultralow-attachment dishes containing the NPCs expansion medium (composed of DMEM-F12, N2 and bFGF). The obtained SNMs were further differentiated into dopaminergic (DA) neurons in the neuron induced medium (composed of DMEM-F12, bFGF-8 and Sonic hedgehog).

### Teratoma formation and analysis

After 5–7 days of culture with eiMEFs, hESCs colonies were suspended with 1mg/ml collagenase IV (Invitrogen, USA) and 1X dispase. Then 5–10×10^6^ hESCs were injected intramuscularly into Severe Combined Immunodeficiency Disease (SCID) mice. Teratomas formed after 2–3 months. Mices were sacrificed and teratoma tissues were dissected into sections and then fixed in 4% PFA. The fixed sections were stained with hematoxylin (Sigma, USA) and eosin (Sigma, USA) and photographed with the microscope of fluorescence inversion microscope (Olympus, Japan). The Institutional Animal Care Committee of Nanjing Medical University approved the experimental protocol.

### Detection of residual ethanol

The ethanol detection kit (Roche) was used to measure the residual ethanol through evaluating the absorbed UV. The supernatant of hESCs culture on culture day 1 to day 7 was tested and the ethanol concentrations were calculated with the formula C = (V×MG/ε×d×v×2×1000)×ΔA[g/l] (V = final volume (l), v = sample volume (l), MG = molecular weight of the substance to be assayed (Da), d = light path (nm), ε = extinction coefficient).

## Results and Discussion

### 10% ethanol-inactivated MEFs (eiMEFs) could well support hESCs proliferation

**Fig 1:**
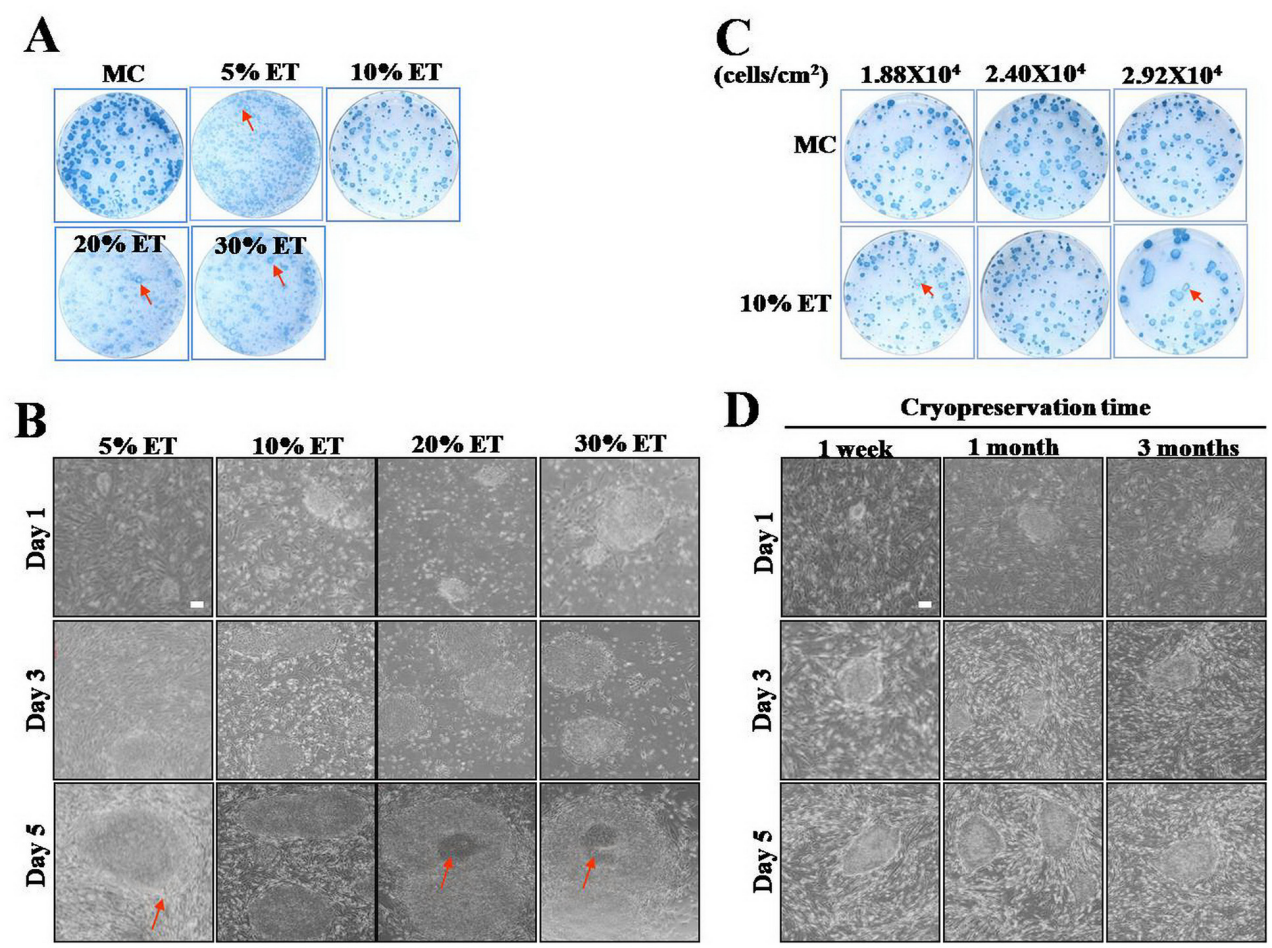
10% ethanol-treated MEFs supported hESCs growth. **A**, Alkaline phosphatase (AKP) staining analysis on hESCs cultured on MEFs treated by different concentrations of ethanol (5%, 10%, 20% and 30%). **B**, The morphology of hESCs cultured on MEFs treated by different concentrations of ethanol (5%, 10%, 20% and 30%) during growth day 1, 3 and 5. **C,** AKP staining analysis on hESCs cultured on ethanol-treated MEFs plated with different densities. **D**, The morphology of hESCs cultured on frozen-thawed eiMEFs. MC, mitomycin C. ET, ethanol. miMEFs, 10 ug/ml mitomycin C inactivated mouse embryonic fibroblasts. eiMEFs, 10% ethanol inactivated mouse embryonic fibroblasts. Red arrows indicate differentiated cells. Scar bar, 100 μm.

Chemical fixed-MEFs are convenient and economical in application to embryonic stem cells maintenance, but such MEFs were not proved to support hESCs growth[29, 30]. Recently, research showed that 70% ethanol (ET) could completely fix MEF and support hESCs growth[31], providing a healthy method for preparing MEFs. Nevertheless, those chemical-fixed MEFs can only be cryopreserved on culture plates[30], but can not be applied through the “frozen-thawed” approach, leading to the limited application. There are reports demonstrated that low concentration of ET can inhibit cell proliferation by impeding DNA synthesis[32, 33], which remind us that MC inhibits cell growth through preventing spindle formation. Thus, here we test if low concentration of ET can inactivate MEFs, and if the ET-inactivated MEFs can maintain hESCs self-renew like the MC has done. In our study, different concentrations of ET (5%, 10%, 20% and 30%) were applied to treat MEFs for 30 minutes and then the treated MEFs were co-cultured with hESCs. Meanwhile MC-inactivated MEFs (miMEF) were set as the control. We found that hESCs tended to differentiating when growing on the MEFs treated by 5%, 20% and 30% ET (Fig 1A and 1B), and there was less hESC colony grown compare to the control according to AKP staining assay (Fig 1A). However, 10% ET-treated MEFs showed strong competence on supporting hESCs proliferation (Fig 1A and 1B). Our further study revealed that MEFs treated by 5% ET retained their proliferation capacity, while most of MEFs treated by 20% and 30% ET were completely fixed (dead) (S1 Fig). Only 10% ET-treated MEFs were still alive but growth-cessation, similar to that of miMEFs (S2 Fig). Therefore, we predicted that 10% ET-treated MEFs might function as miMEFs on supporting co-cultured hESC growth. Hereafter, the MEFs treated by 10% ET for 30 minutes were called as ethanol-inactivated MEFs (eiMEFs).

It is reported that the density of co-culture MEFs crucially affect hESC growth[38]. Next we evaluated the appropriate density of eiMEFs to well support hESCs culture. Three densities of 1.88×10^4^, 2.40×10^4^ and 2.92×10^4^ cells/cm^2^ were compared in our study. Our results demonstrated that hESCs preferred to the eiMEF density of 2.40×10^4^ cells/cm^2^ (Fig 1C), while differentiated on eiMEFs with the density of 1.88×10^4^ or 2.92×10^4^ cells/cm^2^ (Fig 1C, red arrow).

It will be very convenient in application if eiMEF can stand cryopreservation. Here eiMEFs were assessed if they still functioned on supporting hESC growth after frozen and thawed. In our investigation, eiMEF were cryopreserved in liquid nitrogen for 1 week, 1 month and 3 months respectively. And then cryopreservation eiMEFs were thawed, plated onto culture plates to feed hESCs. We found that MEFs thawed from all three storage stages could well support hESCs proliferation (Fig 1D). The result indicated that eiMEFs are convenient in use because large quantity of MEFs could be treated, cryopreserved in a single experiment and be thawed at different time to satisfy occasional applications.

In summary, similar to miMEFs, eiMEFs could effectively support hESCs growth. However, eiMEFs were healthier (low concentration of ET is compatible with animal and human body), more economical (ET is cheaper than MC), and more convenient (only 30 minutes treatment) than miMEFs in application.

### hESCs growing on eiMEFs highly expressed stem cell markers

**Fig 2:**
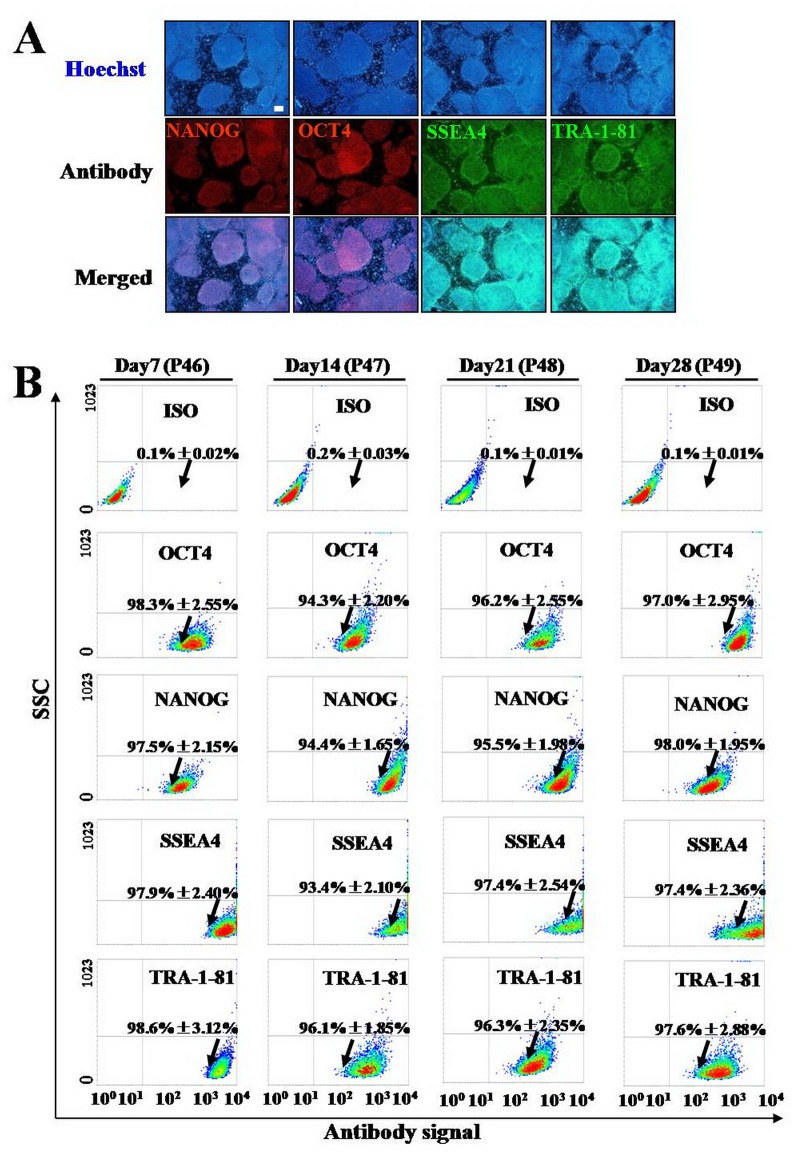
hESCs growing eiMEFs expressed stem cell markers. **A,** Immunostaining showed pluripotent markers of NANOG, OCT4, SSEA4 and TRA-1-81 expressed in hESCs cultured on eiMEFs. Scar bar, 100 μm. **B,** FACS assay showed that during consecutive incubation (day 7 (passage 46), day 14 (passage 47), day 21 (passage 48) and day 28 (passage 49), high percentage of cells positive for OCT4 (98.3%±2.55%, 94.3%±2.20%, 96.2%±2.55%, 97.0%±2.95%), NANOG (97.5%±2.15%, 94.4%±1.65%, 95.5%±1.98%, 98.0%±1.95%), SSEA4 (, 97.9%±2.40%, 93.4%±2.10%, 97.4%±2.54%, 97.4%±2.36%) and TRA-1-81 (98.6%±3.12%, 96.1%±1.85%, 96.3%±2.35%, 97.6%±2.88%) stablely expressed in hESCs cultured eiMEFs.

Though above AKP staining assay showed that hESCs cultured on eiMEFs were undifferentiated stem cells, we further confirmed the cells’ stemness by carrying out FACS and immunofluorescence staining experiments. The Expression level of stem cell markers[39], including OCT4, NANOG, SSEA4 and TRA-1-81 were evaluated. Our immunofluorescence staining results showed that NANOG, OCT4, SSEA4 and TRA-1-81 (Fig 2A) were all highly expressed in growing hESC. To further assess the quality of hESCs growing on eiMEFs, cell population positive for above 4 stem cell markers was evaluated respectively through FACS assay. Four consecutive passages of hESCs (step over 28-day culture) were employed. Our results showed that after 7 (passage 46), 14 (passage 47), 21 (passage 48) and 28 (passage 49) days consecutive culture on eiMEFs, the cell population positive for OCT4 respectively was 98.3%±2.55%, 94.3%±2.20%, 96.2%±2.55%, 97.0%±2.95% (Fig 2B, the second row); the cell population positive for NANOG respectively was 97.5%±2.15%, 94.4%±1.65%, 95.5%±1.98%, 98.0%±1.95% (Fig 2B, the third row); the cell population positive for SSEA4 respectively was 97.9%±2.40%, 93.4%±2.10%, 97.4%±2.54%, 97.4%±2.36% (Fig 2B, the forth row); and the percentage of cells positive for TRA-1-81 respectively was 98.6%±3.12%, 96.1%±1.85%, 96.3%±2.35%, 97.6%±2.88% (Fig 2B, the fifth row). Our data indicated there was very high percentage (>94%) of stem cells in eiMEFs-hESCs culture and the stem cell population almost invaried after long time culture. Therefore, hESCs cultured on eiMEFs retained the undifferentiated state and maintained good quality. In another word, eiMEFs could support the self-renew of hESCs[36].

### hESCs growing on eiMEFs were pluripotent

**Fig 3:**
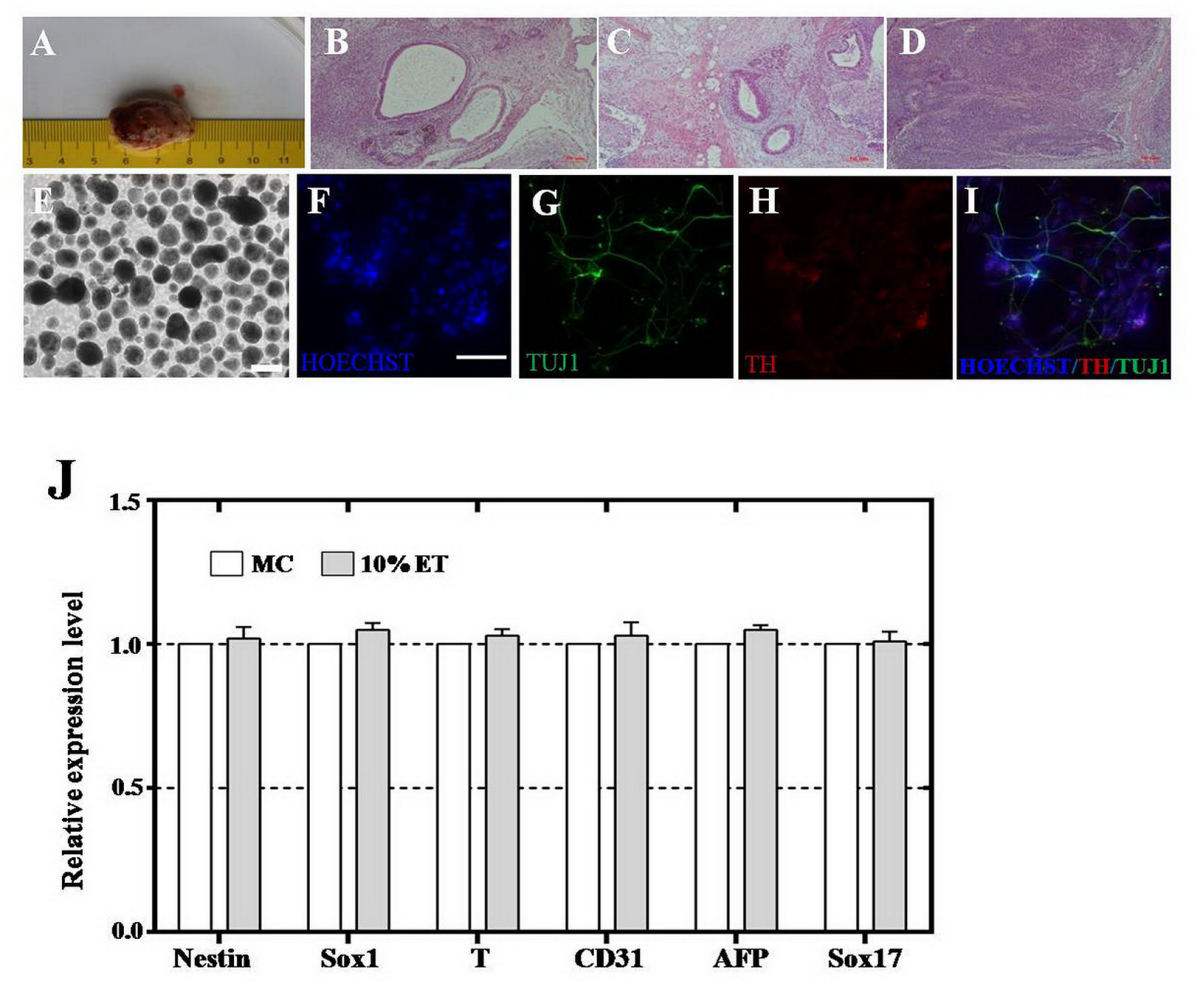
hESCs cocultured with eiMEFs were pluripotency *in vivo* and *in vitro*. **A**, Teratomas formed after hESCs were injected into nud mice for 8 weeks. **B**, Gland tissure in the hESCs-derived teratoma. Scar bar, 50 μm. **C**, Adipose tissue and muscles in the hESCs-derived teratoma. Scar bar, 50 μm. **D**, Epidermal and neural tissues in the hESCs-derived teratoma. Scar bar, 50 μm. **E**, hESCs formed embryoid bodies (hEBs). **F**, Hoechst staining showed cell nucleus (blue). Scar bar, 10 μm. **G**, hESCs-derived immature neurons were indicated by BIII-tubulin (TUJ1, green). **H**, hESCs-derived dopaminergic neuron cells were marked by tyrosine hydroxylase (TH, red). **I**, Picture merged from F, G and H. **J**, Quantitative PCR (qPCR) analysis on the expression level of gene markers of the three-germ layers. *NESTIN* and *SOX1* (ectoderm markers), *T* and *CD31* (mesoderm markers), *AFP* and *SOX17* represented (entoderm markers). There were no statistical differences between the expression level of marker genes in eiMEFs-hEBs and miMEFs-hEBs. miMEFs-hEBs, embryoid bodies derived from hESCs cultured on miMEFs. eiMEFs-hEBs, embryoid bodies derived from hESCs cultured on eiMEFs.

Embryonic stem cells are pluripotency in that they promise to differentiate into all three-germ layers and their derivatives *in vivo* and *in vitro*[40]. Our existing data showed that eiMEFs could support the proliferation and self-renew of hESCs, but the pluripotency of growing hESCs was yet not tested. Here, hESCs cultured on eiMEFs were injected subcutaneously into the 7-week old nude mouse to evaluate the *in vivo* pluripotency. Teratomas formed (Fig 3A) after 2 months transplantation and were isolated, fixed and sliced, then performed hematoxylin and eosin staining (HE). The staining results showed that cell lineages derived from all three-germ layers were generated from the injected hESCs, including gland (endoderm lineage) (Fig 3B), adipose tissue and muscles (mesoderm lineage) (Fig 3C), epidermal and neural tissues (ectoderm lineage) (Fig 3D). Our results suggested that hESCs cultured on eiMEFs maintained their pluripotency of differentiation into all tissues and cells of human body.

Next we assessed the *in vitro* pluripotency of hESCs growing on eiMEFs. We found the hESCs could form embryoid body (EB) (Fig 3E) and day 7 EBs expressed markers of cell lineages derived from all three-germ layers (Table 1), such as *NESTIN* and *SOX1* (represent the ectoderm lineage), *T* and *CD31* (represent the mesoderm lineage), *AFP* and *SOX17* (represented the ectoderm lineage) (Fig 3J). In line with our prediction, all expression levels of above marker genes were comparable to that of EBs generated from hESCs cultured on miMEFs, showing no statistical differences. We further confirmed the differentiation potential of hESCs co-cultured with eiMEFs by directly differentiating the hESCs into neurons step by step according to previously report[35]. Our results revealed that the hESCs could sequentially differentiate into BIII-tubulin (TUJ1) positive neuron progenitor cells (Fig 3G), and tyrosine hydroxylase (TH) positive dopaminergic (DA) neuron cells (Fig 3H).

Our results indicated that hESCs feeded by eiMEFs remained their pluripotency, could form teratomas *in vivo* and differentiate into all three-germ layers and their derivatives *in vitro*, suggesting that eiMEFs were reliable in culturing hESCs.

### No residual ethanol in eiMEFs

It is well known that maternal exposure to alcohol will affect the development, especial the nerve system development of conceived fetus[41]. Accordingly, it is reported that larger colonies of undifferentiated hESC exposed to 20 mM ethanol will increase apoptosis and decreased glial fibrillary acidic protein (GFAP) expression in later differentiation progress, indicating that ethanol reduced astrocyte differentiation potential[42]. There is also reported that 20 mM ethanol exposure altered the proliferation and differentiation of hESCs-derived neurospheres[43]. Recently, research showed that 1.0% (0.22M) ethanol exposure could affect the expression of key pluripotency markers in ESC, made the ESCs loss their pluripotency[44]. And hESCs treated with 0.1% (22mM) ethanol induced statistically significant changes to metabolite abundance in human embryoid bodies, neural progenitors and neurons[45].

In theory and practice, ethanol in cells can be wiped off by washing because ethanol enters into or goes out cells through free diffusion. Ethanol might be retained in eiMEFs if washing incompletely. The residual ethanol would be released into hESC culture media after long time incubation and then affect the proliferation, apoptosis and differentiation of co-cultured hESC. Thus, it is necessary to detect if there is residual ethanol in eiMEFs.

**Fig 4:**
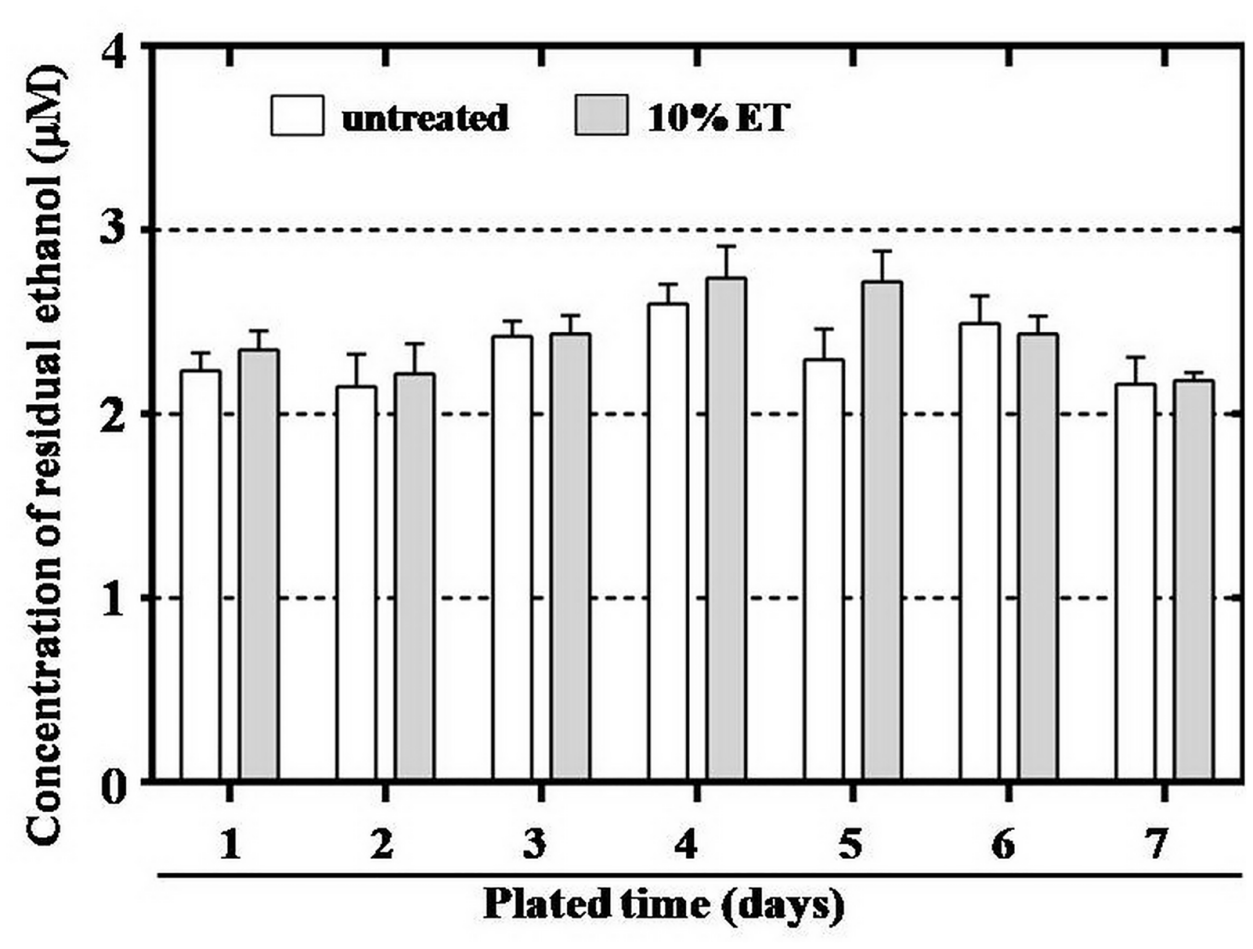
No residual ethanol detected in old culture media harvested from eiMEF culture. Old culture media was harvested at day 1, 2, 3, 4, 5, 6 and 7 after MEFs plated. The concentrations of residual ethanol in harvested media were measured by ethanol detection kit. Negligible, background levels of ethanol were detected in both eiMEFs group and untreated MEFs group. And Statistical analysis showed there was no significant difference between the two groups.

In our study, hESC were cultured for 5–7 days before passage and culture media was changed every day. Thus the old media from eiMEF culture and untreated-MEF, culture at culture day 1 to day 7 were harvested respectively and assessed the concentration of ethanol (CE) with an ethanol detection kit. Our results showed that CE in the old media from eiMEF culture was 2–3μM (Fig 4). There was no statistical difference observed between different harvest time points. Likewise, when CE in the old media from eiMEF culture was compared to that from untreated-MEF culture, no significant difference was determined. Further more, the peak concentration of 3μM was more than six thousands of folds below the harmful concentration of 20mM[42]. Our results indicated that there was no residual ethanol in eiMEFs, suggesting that eiMEFs were safe in hESC culture. And the negligible “ethanol” detected in both old culture media might come from cell metabolism because the ethanol detection kit indirectly measures ethanol concentration based on the concentration of NADH (Nicotinamide Adenine Dinucleotide plus Hydrogen) which can be generated during cell growth[46].

In this study, eiMEFs were developed and applied to maintain hESCs culture. We confirmed that eiMEFs could well and safely support the proliferation (Fig 1) and self-renew of hESCs (Fig 2). In addition, hESCs growing on eiMEFs retained their pluripotency in that they could differentiate into cell lineages derived from all three-germ layers *in vivo* and *in vitro* (Fig 3). Meanwhile, hESCs cultured on eiMEFs kept the normal karyotype (S3 Fig). Moreover, eiMEFs were effective to support the growth of other two hESC cell lines (CCRM 1, CCRM 23) established in our own laboratory (S4 Fig). This data suggested that similar to miMEF, eiMEFs can be broadly used in hESC culture. Attractively, if clinical grade ethanol was applied to inactivate human-derived MEFs (hMEFs), then the obtained eihMEFs could be directly used to support the growth of clinical grade hESCs. Considering above advantages, it can be summarized that eiMEFs are safe, convenient, economical feeder cells, would be applied broadly in hESCs culture.

However, how eiMEFs work to support the proliferation and self-renew of hESCs is unclear. Joddar *et al*. suggest that the cell-formed extracellular matrix-derived substrate support the proliferation and self-renew of hESCs[47]. There are reports implicated that ethanol can inhibit cell proliferation through decreasing DNA synthesis[32, 33]. In our study, eiMEFs were still alive (S2 Fig) but no obvious proliferation, thus were similar to miMEFs. Therefore, we predict that eiMEFs may function through the same mechanism on which miMEFs do: growth cessation cells provide smooth surface with nutrient-rich extracellular matrix[48–53] for hESC adhesion, and meanwhile secret necessary growth factors for hESCs growth[32]. Further investigations are required to confirm our hypothesis.

In summary, our data here indicates that eiMEFs are able to support hESCs proliferation, self-renew and meanwhile remain hESCs pluripotency. The eiMEFs will promote hESC-based research in future.

## Supporting Information

S1 Fig Phenotype of 5%, 20% and 30% ET-treated MEFs. hESCs were co-culture with 5%, 20% and 30% ET-treated MEFs. A and B: After treatment, both at the plating density of 1.88×10^4^ and 2.92×10^4^ cells/cm^2^, 5% ET-treated MEFs kept growing. at last forming compact cell lays (red arrow) and co-cultured hESCs differentiated. C and D: Most of 20% ET-treated MEFs dead and small number of cells left (red arrow), co-culture hESCs differentiated. E and F: Most 30% ET-treated cells dead and small number of cells left (red arrow), and co-culture hESCs differentiated. ET, ethanol. Scar bar: 100 μm. MEFs, mouse embryonic fibroblasts.
(TIF)

S2 Fig The percentage of alive MEFs after MC or 10% ET treatment. MC- and 10% ET-treated MEFs with determined number were plated and cultured. The alive cells were harvested and counted respectively at day 1, 3, 7 and 14 after plated. The percentage of alive cells was calculated by compared the number of alive cells to the number of initially plated cells. The percentage of alive cells in 10 ug/ml MC treatment was not changed during the 4 times points checked. And the percentage of alive cell in 10% ET treatment showed a small increase at day 3, 7 and 14, but no significant difference was determined. MC, mytomycin C. ET, ethanol. MEFs, mouse embryonic fibroblasts.
(TIF)

S3 Fig The karyotype of H9 was normal after long-term culture on eiMEFs. Standard G-band chromosome analysis was performed to identify the karyotype of hESCs cultured on eiMEFs. Normal chromosome number and normal G-band pattern were observed. eiMEFs, 10% ethanol-treated mouse embryonic fibroblasts.
(TIF)

S4 Fig eiMEFs supported CCRM1 and CCRM 23 growth. hES lines of CCRM 1 and CCRM 23 at passage 39 were cultured on eiMEFs or miMEF. The morphology of hESCs at culture day 1, 3 and 5 were observed and photographed under microscope. eiMEFs, 10% ethanol-treated mouse embryonic fibroblasts. miMEF, 10 ug/ml mitomycin C-treated mouse embryonic fibroblasts. Scar bar, 100 μm.
(TIF)
