# Supplementary figures and images for: Ethanol Inactivated Mouse Embryonic Fibroblasts Maintain the Self-Renew and Proliferation of Human Embryonic Stem Cells
Source: PLoS One. 2015 Jun 19;10(6):e0130332. doi: 10.1371/journal.pone.0130332 (PMC4474813; doi:10.1371/journal.pone.0130332)

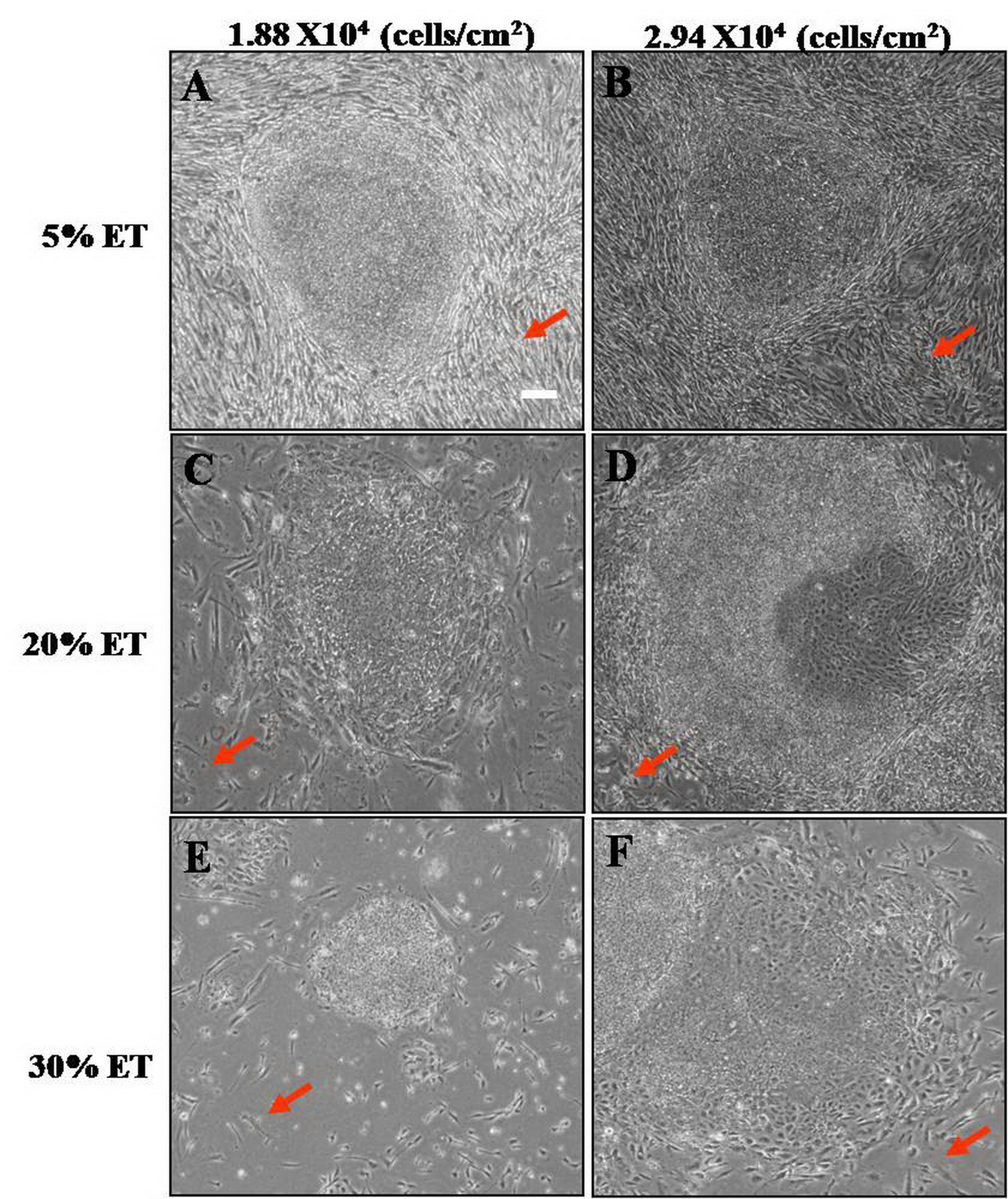

Supplement: S1 Fig — hESCs were co-culture with 5%, 20% and 30% ET-treated MEFs. A and B: After treatment, both at the plating density of 1.88×10^4^ and 2.92×10^4^ cells/cm2, 5% ET-treated MEFs kept growing. at last forming compact cell lays (red arrow) and co-cultured hESCs differentiated. C and D: Most of 20% ET-treated MEFs dead and small number of cells left (red arrow), co-culture hESCs differentiated. E and F: Most 30% ET-treated cells dead and small number of cells left (red arrow), and co-culture hESCs differentiated. ET, ethanol. Scar bar: 100 μm. MEFs, mouse embryonic fibroblasts. (TIF) [file pone.0130332.s001.tif]

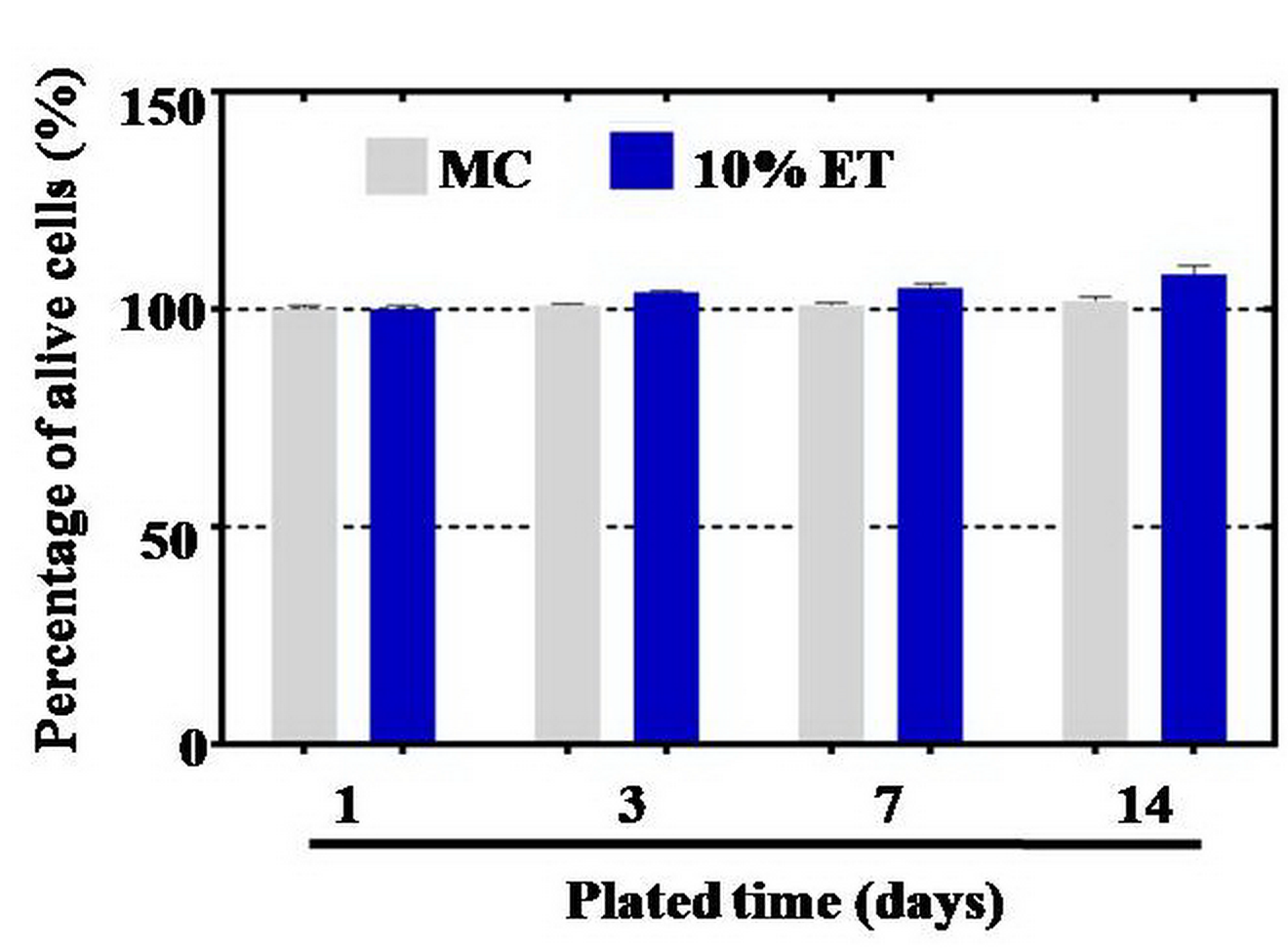

Supplement: S2 Fig — MC- and 10% ET-treated MEFs with determined number were plated and cultured. The alive cells were harvested and counted respectively at day 1, 3, 7 and 14 after plated. The percentage of alive cells was calculated by compared the number of alive cells to the number of initially plated cells. The percentage of alive cells in 10 ug/ml MC treatment was not changed during the 4 times points checked. And the percentage of alive cell in 10% ET treatment showed a small increase at day 3, 7 and 14, but no significant difference was determined. MC, mytomycin C. ET, ethanol. MEFs, mouse embryonic fibroblasts. (TIF) [file pone.0130332.s002.tif]

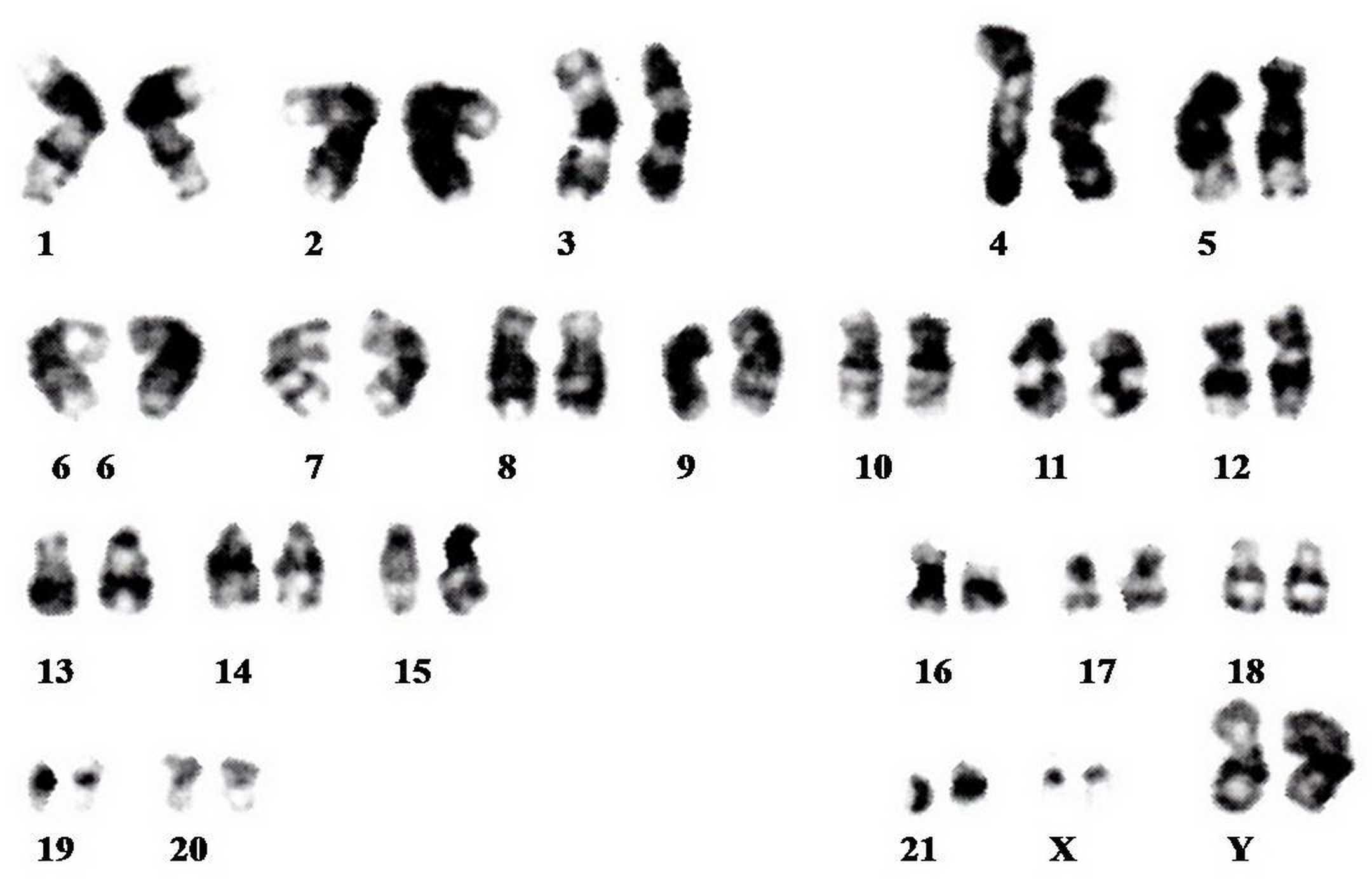

Supplement: S3 Fig — Standard G-band chromosome analysis was performed to identify the karyotype of hESCs cultured on eiMEFs. Normal chromosome number and normal G-band pattern were observed. eiMEFs, 10% ethanol-treated mouse embryonic fibroblasts. (TIF) [file pone.0130332.s003.tif]

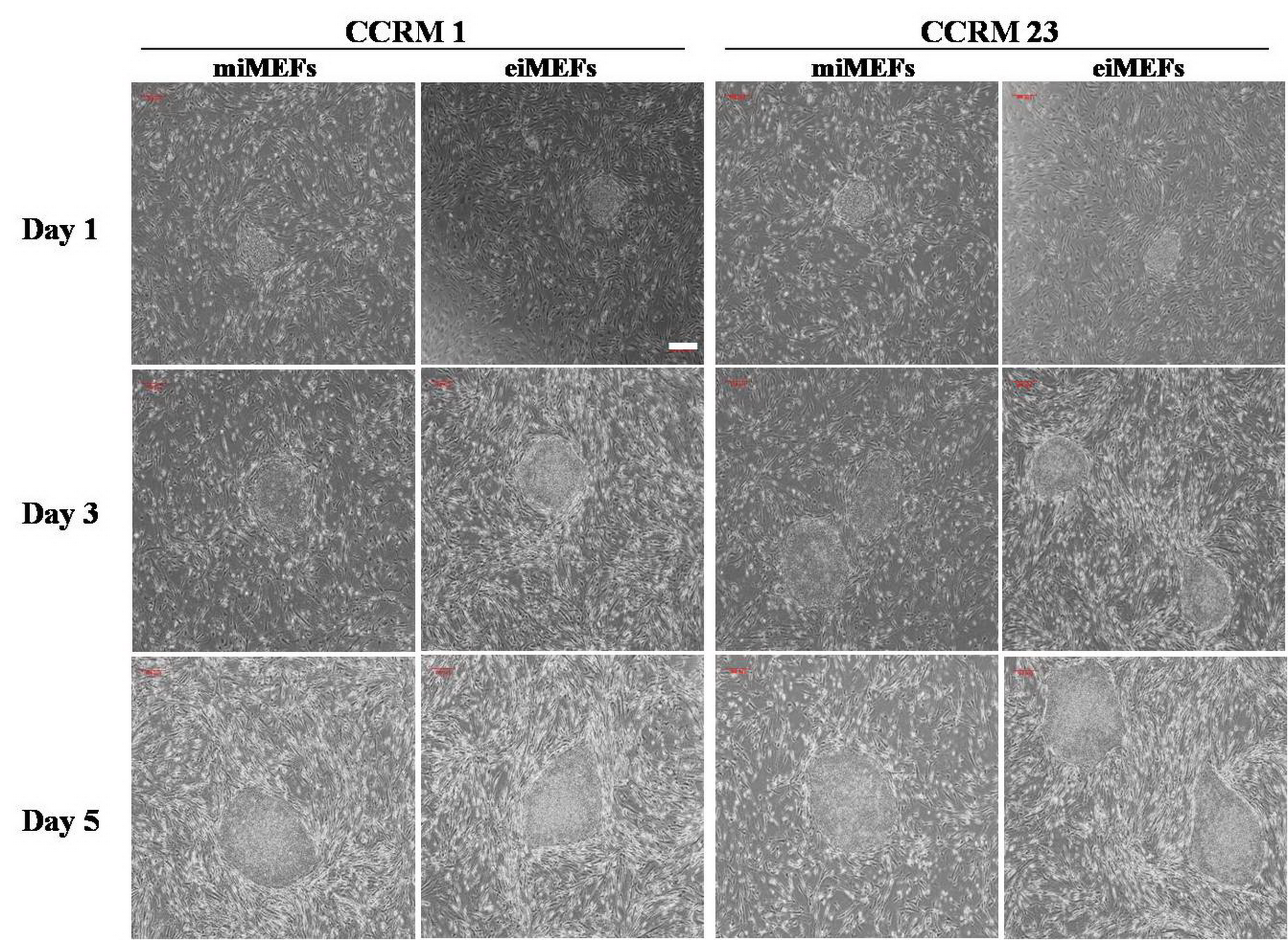

Supplement: S4 Fig — hES lines of CCRM 1 and CCRM 23 at passage 39 were cultured on eiMEFs or miMEF. The morphology of hESCs at culture day 1, 3 and 5 were observed and photographed under microscope. eiMEFs, 10% ethanol-treated mouse embryonic fibroblasts. miMEF, 10 ug/ml mitomycin C-treated mouse embryonic fibroblasts. Scar bar, 100 μm. (TIF) [file pone.0130332.s004.tif]
